# Governing Ethical AI Transformation: A Case Study of AuroraAI

**DOI:** 10.3389/frai.2022.836557

**Published:** 2022-02-10

**Authors:** Jaana Leikas, Aditya Johri, Marko Latvanen, Nina Wessberg, Antti Hahto

**Affiliations:** ^1^VTT Technical Research Centre of Finland Ltd., Tampere, Finland; ^2^Department of Computer Science, Aalto University, Helsinki, Finland; ^3^Department of Information Sciences & Technology, George Mason University, Fairfax, VA, United States; ^4^Digital and Population Data Services Agency, Helsinki, Finland; ^5^Ministry of Finance, Helsinki, Finland

**Keywords:** artificial intelligence, ethics, governance, co-design, public administration (generally)

## Abstract

How can the public sector use AI ethically and responsibly for the benefit of people? The sustainable development and deployment of artificial intelligence (AI) in the public sector requires dialogue and deliberation between developers, decision makers, deployers, end users, and the public. This paper contributes to the debate on how to develop persuasive government approaches for steering the development and use of AI. We examine the ethical issues and the role of the public in the debate on developing public sector governance of socially and democratically sustainable and technology-intensive societies. To concretize this discussion, we study the co-development of a Finnish national AI program AuroraAI, which aims to provide citizens with tailored and timely services for different life situations, utilizing AI. With the help of this case study, we investigate the challenges posed by the development and use of AI in the service of public administration. We draw particular attention to the efforts made by the AuroraAI Ethics Board in deliberating the AuroraAI solution options and working toward a sustainable and inclusive AI society.

## Introduction: AI and Sociotechnical Change

AI has a great promise to offer solutions to the problems of humankind. It will ultimately change the structures of society and everyday lives of people in a profound way. In this development, the human focus should be on values that emphasize humanity and the understanding of the social impact of AI.

AI is difficult to define, and no single universally accepted definition has been established within the scientific community. We often hear talk of weak and strong AI, the latter of which has not even been implemented in practice. AI systems are built on a variety of methods. Central areas to AI include machine learning, natural language processing, computer vision, speech recognition, planning and scheduling, optimization, robotics, and expert systems (Holton and Boyd, 2021; Pietikäinen and Silvén, 2021). To achieve a given complex goal, AI systems observe the environment, acquire data, and make inferences and decisions based on the data and information. They collect and process both structured and unstructured data and make inferences based on this data.

Holton and Boyd ask where the people are in these elements of AI. The answer is not straightforward, but a great fear is that the human viewpoint remains distant in AI applications. Holton and Boyd (2021) argue that “while human consciousness retains distinctive features, these do not support an anthropocentric perspective on human–machine interactions.”

One of the key questions is whether we accept the benefits of AI if we do not totally understand the impacts of the technology on society and citizens. This is something that the governments need to deal with: to see that the potential of AI can flourish. That is, to help individuals prepare, understand, accept and embrace AI, and maybe even to refuse the use AI. Beal et al. (2016) list the challenges in building AI systems as follows:

1. Difficulty in capturing expert knowledge: much of the knowledge held by experts is not explicitly written down anywhere.
2. Structural barriers to knowledge exchange: designers may not be able to access or share their knowledge due to cultural, organizational, or legal barriers.
3. Gaps in scientific knowledge: AI techniques can only produce effective improvement or automation of processes carried out by humans if the processes are well-understood in the first place.
4. Rapidly advancing knowledge and methods: how the system performance is ensured.
5. Cost of adoption vs. rapid advance: how the system performance is controlled.

According to this list of challenges it seems that the most demanding goal is to understand the system and to gain the knowledge to control its performance.

A human-centric AI transformation in the governance of public services requires participation from the public to ensure that the governance works as intended. As governance becomes more complex, which is the case with most developed societies, it is often hard in democracies to engage the public even in the basics of governing processes such as voting. This is especially true for systems that interact with or interface with the public at some level as it is not always obvious to the public how the process works or they might not have the expertise needed to engage with a service. This problem is further complicated by the increasingly abstract nature of the digital world where there are even more layers of complexity that can be used to hide what is going on. In addition, AI introduces another layer of complexity as even many experts lack a clear understanding of the workings of many systems that are in use.

This whole process also assumes that there is a basic common understanding of the ethical and moral issues involved and this might not be true given the diversity within the population and divisions between the technology-haves and have-nots or those who can use technology more effectively than others. What practices might be address these issues in the future? What kind of a cultural change is necessary to create a mechanism for this? We want and need new or novel data and need to redefine relationships with those who are being governed. It is also about expertise and who has it and what their motivations are for creating or designing a new system. In other words, a lot of this works on trust and how we keep the governance systems trustworthy.

In addition, as AI evolves, we need a new kind of ethical reflection. This means constant ethical self-examination and vigilance alongside AI development. Ethical experts, scientists, technology developers, and other relevant stakeholders need to be brought together to deliberate the ethics of AI in a multidisciplinary way.

The purpose of this paper is to draw particular attention to the ethical steering efforts in developing an AI system for public administrations and finding a sustainable and inclusive solution. The paper (1) describes the way the Ethics Board of AuroraAI operates, and (2) raises essential ethical questions about the design of AI for citizens under the guidance of a public administrator.

## The Ethical Use of AI in the Context of Public Services

### The Role of Public in the Governance of AI

Research has shown that although the use of AI in government has many potential benefits, it also creates challenges such as reducing citizens' trust in government (Al-Mushayt, 2019; Gupta, 2019; Sun and Medaglia, 2019), including citizens' trust in the decisions made by government (Sun and Medaglia, 2019). This distrust often emerges due to violation or perceived violation of privacy and/or through a perception of lack of fairness in the outcomes of AI systems for public governance (Kuziemski and Misuraca, 2020). In particular, the challenge in the use of AI is a lack of transparency of black-box AI systems whereby it is difficult to assign responsibility and accountability for decision-making (Dignum, 2018; Wirtz et al., 2018). Overall, the reality of using AI raises the stakes for AI use in the public sector as the risk is heightened in case of system failures as it will result in negative implications for governments and society.

In theory, as AI use increases across aspects of society the public sector implementation should become better as well. In practice, this is difficult because whereas the private sector has more leeway to experiment with AI practices, the public sector has to focus on maximizing public value and public good as opposed to other outcomes (Fatima et al., 2020). In other words, the penalty for causing harm can be very high in the public sector. Consequently, the use of AI in the public sector needs to be transparent to the extent possible, in effect to gain citizens' trust (Bryson and Winfield, 2017), and to comply with the need for regular scrutiny and oversight (Desouza et al., 2020, p. 206). Finally, the complexity of using AI in the public sector arises from a diverse set of stakeholders who are involved and who often have competing interests and agendas (Desouza et al., 2020). These challenges, coupled with the rapid development of AI has created a situation where public services and administration find it difficult to keep up (Wirtz et al., 2018, p. 826) and policymakers need to pay more attention to the potential threats and challenges posed by AI. These concerns call for design and implementation of better governance structures and policy development but also for rethinking the role of the public, and the challenges they face, in working with AI-driven public services.

The need for increased public engagement in the deployment and even in the design and development of AI services has been well-recognized by a range of organizations. Engagement with the public and raising public awareness serves two major functions. First, a transparent debate builds trust by involving the public, and second, better outcomes for design and implementation can be reached through public engagement. Organizations working on this issue have come up with guidelines on facilitating and including public voices. As an example, the RSA has outlined three issues that are particularly relevant for public deliberation: transparency and explainability; agency and accountability; and fairness^1^. Whereas, the RSA's work is focused on the short term, one time, engagement, others have argued for longer-term and ongoing engagement with the public. A sustained debate is important to influence policy decision-making and to ensure a more democratic and trustworthy process from the public's perspective. Of course, as others have pointed out, sustained engagement is important but not without its challenges including the pace of change of technology, complexity of the technology, and the inability to predict AI's trajectory and consequential potential impacts and the benefits, risks and harms which may result. These challenges are reinforced given the stakeholders involved.

The organization *Involve* ran a series of roundtable discussions with a wide range of stakeholders working with AI and with a clear interest in public engagement in AI^2^. They found that there is a need to develop a shared understanding of what public engagement with AI and governance means, and how it can be achieved. They argue that engaging the public with different framings and questions could make it difficult for policy makers to make sense of the findings, diminishing the impact of public perspectives on policy decisions. They argue that those “making decisions about the design, deployment, and use of AI need to work collectively to develop more of a shared understanding of how the public should be involved in their decisions.” They concede that even though a shared understanding might not be achieved, there is value in bringing people together to voice their perspectives and acknowledge their differences. They make recommendations for public participation starting with creating “focused engagement” to overcome the generally diffuse and unfocused conversation around AI. For instance, engagement around facial recognition, credit scores, etc. is more preferred than overall generic discussions. It is also important that those working with AI “demonstrate possible futures and do more to deconstruct the hype surrounding AI.” This is needed to illustrate trade-offs and explain where AI is and is not in people's lives to increase awareness and understanding. Third, it is important to identify which publics are important to engage with on an issue. They argue that there are multiple publics and communities with multiple perspectives and views and therefore, identifying and engaging the relevant perspective is important.

### Data Ownership and Privacy

Data openness and issues of privacy are important but not always easy to implement given the expertise that is needed to put them into practice. If we want “humans” or the public to be able to make use of these provisions or be able to safeguard their own privacy, then we need to create not just **awareness** but also the **ability** to engage meaningfully. This is a challenging goal as it would require imparting a lot of education and training.

In terms of data and privacy issues, the challenges faced by the public are well-documented and range from the unethical use of data (Gupta, 2019), lack of data privacy (Valle-Cruz et al., 2019), to challenges with data security (Toll et al., 2019). Public users are worried about novel challenges to the privacy of data in AI systems for governments (Fatima et al., 2020) and how to curtail privacy violations (Kuziemski and Misuraca, 2020).

The challenge to creating AI-driven services for the public come largely from what Zuiderwijk et al. (2021) refer to as the skills challenge. In their review, they document different aspects of this problem including limited knowledge about machine learning and AI among the staff (Ojo et al., 2019). Differential skills levels of people in the organization based on their function and background, inhibits cross-sectoral collaboration around AI (Mikhaylov et al., 2018). Researchers have also documented a lack of in-house AI talent (Gupta, 2019; Sun and Medaglia, 2019) coupled with gaps in education for highly technical skills (Montoya and Rivas, 2019). Overall, a lack of expertise (Al-Mushayt, 2019) coupled with increased demand for a limited number of AI experts (Wirtz et al., 2018) has resulted in the need to train more people.

The challenges of the ethical use of AI in the context of public services also stems from the fear that administrative discretion may be misused (Aoki, 2020), the dependency of people on AI that would be created as increasingly services start using it (Ben Rjab and Mellouli, 2018), and the possible severe unfairness of public services that might result.

### Challenges to Public Understanding of and Engagement With AI Ethics

There are challenges and often misconceptions of AI that need to be corrected while the symbolism surrounding it leads to myths that need to be debunked. What is novel here is how much the public are expected to learn to be able to engage with the services—about how it works, about the technology and AI in general.

What does ethical AI mean for people? Given the rise in problems and challenges that are being created with the rise in the use of AI across sectors of society, many organizations, both public and private have come up with and published frameworks, principles, and guidelines on the ethical use of AI (Jobin et al., 2019; Hickok, 2020; Zicari et al., 2021). These include, e.g., the Asilomar AI Principles (Asilomar Conference, 2017), the guidelines of the European Group on Ethics in Science and New Technologies (EGE)^3^, the European Commission's High-Level Expert Group on Artificial Intelligence (ALTAI)^4^, and AI4People (Floridi et al., 2018), to mention but a few. Although the use of guidelines has increased and is finding favor with organizations, they are limited in the sense that most of them have been developed through discussions and input from industry and academic experts, and rarely include feedback from users, including citizens.

Drobotowicz et al. (2021) conducted a qualitative study to investigate citizens' requirements for trustworthy AI services in the public sector. They interviewed 21 Finnish residents and conducted a design workshop on four public AI services which included cases in housing, health, education, and social service domains. Their study was part of a larger project that aimed to provide ethical guidelines for AI usage in the public sector in Finland. They found that **transparency** was a critical requirement for trustworthy AI services from the perspective of citizens. Participants wanted to know the purpose of a service—why it existed and what impacts it would have on them and others—and this was especially important when the benefits of a service were not clear. Participants also expressed an interest in knowing more about the data including data sources and collection and if consent was provided for data use. Finally, **privacy** was another topic participants were keen to know about and they wanted to know where the data would be stored and who would have access. Consistent with findings from Chazette et al. (2019), who found that their respondents wanted service-result explanations, the results from this study also show that for participants understanding what was taking place and why was more important than how (the process). Overall, the findings from this study are interesting and relevant as they show that AI transparency and AI **explainability** are tightly related. Interestingly, few participants in Drobotowicz et al.'s study brought up the topics of bias and fairness, even though these are some of the most common issues found in AI guidelines and principles (Jobin et al., 2019) suggesting these topics might not be known among non-specialists. The other issues that came up were the need to interact with a person to discuss a service, a certain level of **control** over their data, **consent** before data collection or sharing, and the ability to choose which data could be used and withdrawn at any point.

Jobin et al. reviewed 84 ethical AI guidelines proposed by industrial and scientific institutions, 10 of which targeted the public sector. They found five principles that were present in over half the guidelines: (1) transparency, which aims to increase system explainability, interpretability, or disclosure; (2) **justice and fairness**, which are connected to mitigating bias and discrimination and enabling challenge or redress; (3) **non-maleficence**, which focuses on system security and safety; (4) **responsibility**, which is often presented alongside accountability and refers to legal liability and integrity; and (5) privacy, which mostly relates to data protection and data use and is presented both as a value and a user right. In relation to the public sector, the Alan Turing Institute (Leslie, 2019) have articulated a set of guidelines in three parts: (1) support, underwrite, and motivate **values** for a responsible data ecosystem; (2) fairness, accountability, **sustainability**, and transparency principles for designing and using services; and (3) a process-based **governance framework** to operationalize these guidelines. The Harvard ASH center (Mehr, 2017) also has a set of guidelines that explores the use of IA for citizen services, and they suggest six strategies for the government: (1) **make AI part of a citizen-centric program**, (2) **solicit citizen input**, (3) **build on existing resources**, (4) **be data-prepared** and tread carefully with privacy, (5) **mitigate ethical risks** and a**void AI decision making**, and (6) **focus on augmenting employees**, not replacing them.

## AI as Part of a Citizen-Centric Program: Case AuroraAI

### Supporting Citizens in Different Life Events

Finland has been amongst the forerunners in developing AI. Finland's national artificial intelligence program AuroraAI (AuroraAI)^5^ aims for a people-oriented society in which public and private organizations cooperate to help ensure people can deal with life events easily and conveniently at all stages of their lives. AuroraAI points citizens to potential public services. The AuroraAI program aims to be a service network that interconnects services so that they can support and interact with each other, and to implement AI innovations based on the key life events of different human transitions (family situations, progression to education), using flexibly interacting multi-stakeholder ecosystems (SAIP, 2019) and building new service chains that automatically support life event transitions. Service ecosystems use AI solutions to develop entirely new types of services that are tailored to people's personal life situations and to what businesses can offer. This will give citizens better access to personalized services based both on the personal data they provide (MyData; collected for example through personal smart health devices) and on population-level data. Individuals will be able to produce data themselves and access it in usable digital format from the data controller.

As an ambitious program, AuroraAI aims to build a national digital infrastructure where society's current service structures are transformed into **unified service entities using AI**. It is hoped that this will improve the capacity of organizations to strengthen people's wellbeing by providing services for people's various life events intelligently, in collaboration between different service sectors and providers, and by using emerging technologies in a people-oriented way. In addition, this can reduce service costs and create opportunities to integrate public and private services. The Finnish government's policy summarizes the objectives as follows. “Success in achieving the goal of public services requires the interconnection of public organizations (AI-Aurora network) to interact with services from other sectors through AI. The AuroraAI program aims to create a network of services that interconnect services so that they can support and interact with each other” (SAIP, 2019).

The program simultaneously intertwines governance and policy change, regulatory issues, technological innovation in a multi-vendor environment, the pooling of private and public sector interests, data-based modeling of individual and population situations, new procedures and actors' roles in the production and management of public services, as well as increasing the overall wellbeing of the individual. One of the key principles is user-given, unvalidated and anonymous data, through which the sharing of a person's own information with the AuroraAI network would take place. The model is expected to lead changes in the authorities' operational model. To support these changes, it is intended that the model will operate as ethically as possible. The ultimate grand idea is to promote a digital Finland, where **everyone can use advanced services on their own terms and under their own autonomy with the opportunity to participate in the development of services**. To make this goal possible, the program follows and implements the ideas and methods of **open co-innovation**.

People's lives are composed of all kinds of events, such as starting daycare and school, building a family, working life, taking care of family members, and retiring. The idea of the AuroraAI program is that with the help of AuroraAI, people's ability and desire to take care of their own wellbeing will improve, as people and services meet better with the help of AI.

**Figure 1:**
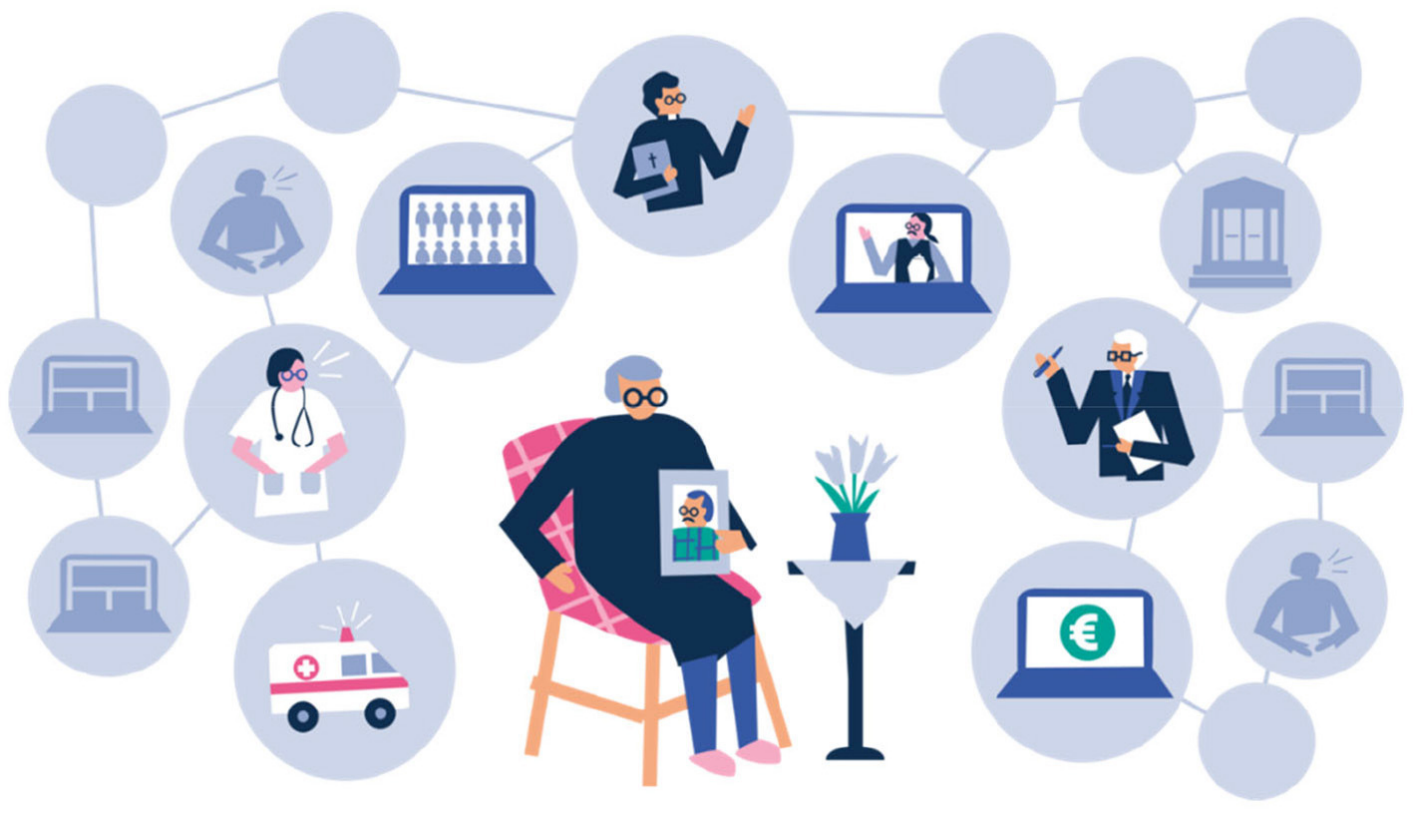
The AuroraAI program will support citizens throughout their life events (Ministry of Finance).

In AuroraAI, people are divided into different clusters based on their multidimensional wellbeing (Figure 1). This **life-event view** is needed to contextualize the need for AI-based services and thus help the design, and eventually to provide seamless service paths for citizens. With the help of clusters, AuroraAI enables the targeting of suitable and timely service packages for a person's individual life events and situations. A life situation can be considered as a state of a state machine, which consists of a nearly infinite number of life situations and transitions between them (life events). In this context, a “state” covers all the information Aurora services have stored about the user in a distributed, anonymous manner. A change of this data yields a state transition. AuroraAI aims to facilitate these transitions by orchestrating optimal micro service combinations from an available pool to meet users' personal needs. People with similar data attributes are considered to reside, partially in similar life situations and therefore to benefit from similar service combinations.

The AuroraAI program has several focal points and dimensions:

- On the technology and data side, the Aurora Platform:
  ◦ connects different services together intelligently in an AI powered network that removes the public/private fence; this allows for smarter, user-centric service findability, and provision;
  ◦ makes possible the development of DigiMe, a data-based digital profile of a real person (services that are based on personal data provide users with better understanding and control over their welfare and the ability to activate services in real time). DigiMe is intended to be used in situations where the connection between a real-world person and their digital persona needs to be made invisible. The user aggregates their personal data and produces a summary that can be processed by the network without being linked to the user's source data. The development and controlled testing of such a concept is important for the privacy of the users and their trust in the system;
  ◦ connects a user's intentions and needs (identified from personal info and attributes) with semantically harmonized and machine-readable service descriptions to generate personalized service recommendations; and
  ◦ forms the framework for the AuroraAI account, with profile management and service sessions transfers between different providers.
- On the side of user functionalities, the intended impacts and vision for future society include:
  ◦ personalized wellbeing estimates and service recommendations (using the DigiMe);
  ◦ data-based “shared wellbeing snapshots” and service needs predictions for various population clusters and down to individuals;
  ◦ sharing of personal life situation info and attributes to the Aurora Platform in order to get service recommendations;
  ◦ AuroraAI working as your personalized “map and compass” that helps you reach your own specific goals or “states in life”; and
  ◦ service ecosystems for various population clusters and social issues, based on masses of individual data gathered through interviews, questionnaires and web surveys, with a **human-centric, AI-powered society with predictive capabilities** as the ultimate goal.

The program ranges from smart service findability and provision that would help a person in a specific situation or service need, to the DigiMe, with its dimensions of individual wellbeing and empowerment, and finally to a new kind of AI-driven society where all service production is a reflection of data-analyzed states of wellbeing and service needs of the population and individuals.

From an individual and societal point of view, **human-centricity** in AuroraAI means that AuroraAI enables services to be more accessible, effective and better-targeted at people's real needs. It aims to put an end to the way people are passed between agencies in order to enable people to manage their lives more easily.

From a technical point of view, **human-centricity** in AuroraAI means taking account the person as a whole upon the provision of the services. This is opposite to a business-centric view, where a person is seen as a customer, and where, for example, the tax administration, a national church, employment service, or a golf club each has a different view of the user, only providing services in their own context and from their own organizational silos. This specialization works in terms of efficiency, but for the end-user it offers only a suboptimal solution to any complex life situation: there is rarely any single service or entity that can help the user with a wide range of different problems. In AuroraAI, it is assumed that providing a technical solution to connect current, siloed services together, combining the viewpoints of several organizations and providing *in situ* service combinations from several cross-sectoral organizations, truly results in more holistic help.

The use of networked micro services makes it possible to better adapt to individual user needs, and AuroraAI network learns the best possible combinations from historical data. Since loosely coupled services can be developed independently and can be re-usable in a multitude of situations, this leads to advantages that have traditionally only been seen in SOA software architectures—not to mention cost savings. It is worth mentioning that the AuroraAI service referred to here are a much broader concept than in the software context: a local swimming pool can exist as an AuroraAI service, connected to the AuroraAI network in the same way as data sources or software. At first, this seems very counter-intuitive. However, a common API between all AuroraAI-enabled services makes this possible and leads to true digitalization: a combination of digital and non-digital services interacting with each other.

### Ethics Board as a Tool for Ethical Deliberation

In autumn 2020, the AuroraAI program set up an Ethics Board with the aim of helping AuroraAI's governance to move in a human-centric, ethical and responsible direction and to verify the use of AI for human wellbeing. This was 2 years after the official start of the program whose concept had already been in development since the summer of 2017 at the Ministry of Finance and an emerging public-private multi-organization network of interested parties and companies.

The Board decided to follow a **forward-looking, proactive ethical deliberation process**, which includes participatory ethical design and aims not only at identifying problems but also at finding ethically sustainable solutions for implementation (Sengers et al., 2005; Stahl et al., 2010).

The key elements of the process are:

- *Anticipation*: Proactive ethical thinking in the development of design solutions; looking carefully at both the objectives and the potential unintended consequences of deploying an application or a service
- *Involvement*: Involvement of technology users or user representatives and developers in identifying and discussing ethical challenges s in a specified context, and
- *Expertise*: Involvement of experts of ethics, technology, social and behavioral sciences, and law in the discussions.

The size of the Board was decided to be limited to be large enough to accommodate different perspectives and fields of expertise, but small enough to function and communicate as a group rather than a network. Because public engagement as such was perceived as challenging in the development work, it was decided that the Ethics Board would invite organizations that represent the public broadly. That is why the Board is represented not only by experts and researchers, but also by representatives of various non-governmental organizations. Altogether 15 organizations participate in the Board, with representations from two universities, NGOs—Nongovernmental Organizations, ministries, the Association of Municipalities, the Association of Technology Companies, the Technical Research Centre of Finland Ltd. (VTT), the Lutheran Church of Finland, and the Finnish Digital Agency (DVV). The Board's coordinator and secretary is from the DVV. Individual Board members have a range of expertise, extending from knowledge of technology, data and legal issues to minority stakeholders, social sciences, and cultural studies academics.

As all short, medium, and long-term objectives were included in the program, there was a risk that the AuroraAI concept as a whole would be difficult to comprehend and discuss. The program itself is vastly ambitious and includes a range of goals and targets. Thus, it necessarily lays itself open to equally many points of analysis, which are sometimes critical and even biting. One of the main strategic questions was whether the focus should be on macroeconomic (a better national economy through improved welfare and individually empowered citizens) or highly personalized (AI helping users to achieve their own life goals).

Therefore, in order to address the ethical issues effectively enough, the Ethics Board decided to take a systematic approach to the debate. The focus of the work was set on the non-technical dimensions of the AuroraAI program. This included:

- Background assumptions, value base, and social vision,
- Human and social impact,
- Power structures,
- The content of governance transformation,
- The meaning and interpretation of human-centricity,
- Interpretations of foresight and a foresighted society, and
- General application of AI to socio-economic and political issues.

Based on this, the Board held six themed meetings in 2020–2021, focusing on jointly selected, specific issues. These were:

1. The AuroraAI concept, aims and underlying philosophy of the program,
2. The “How Am I Doing” functionality and the DigiMe,
3. Service Recommendation Engine and the technical Aurora Platform,
4. Service Ecosystems,
5. Legal issues concerning (a) personal data and privacy and (b) competition and EU single market issues: commercial service providers in the Aurora Platform and the removal of the public/private service provision barrier, and
6. Equality and non-discrimination.

On the basis of this work, the Board produced a set of concrete, pragmatic recommendations for practical measures to be taken in the program. These measures should enforce the ethical foundation of the program and help to tackle some issues the Board has judged as particularly problematic.

## Key Ethical Elements

The Board received a deep insight into the program's various dimensions, aims and the AuroraAI worldview. It discussed these themes, deliberated on what was learned, and produced two reports for the program leadership containing relevant ethical issues, opinions and suggestions for improvement. Quite a few things were deemed worthy of closer deliberation, analysis, and actions. Some of them appear frequently in AI ethics debates and discussions worldwide, while some are unique to the AuroraAI program. The main issues are presented in the following.

### The Greenlighted Elements and the Dilemma of Doublethink Inside the Program

The Board positively noted the following:

- The goal of **better and smarter service findability** is obviously valuable in an environment of severe information overload. The use of AI to help a citizen find the relevant services or information and service providers in their situation is a worthy target. This includes finding responsibly and ethically sound ways of having personal information enrich the inquiries so that citizens can be given as accurate and relevant selection of services as possible without compromising their privacy and autonomy.
- The goal of creating **service ecosystems for specific life events**, combining several service providers from different sectors in a network and facilitating the automation of complex service processes, was met with warm approval. Almost everyone typically encounters at least one life event, many come with processes that are complex, stressful and sometimes painful. Turning these processes into smooth, digitally driven, AI-boosted events that ease the burden of both citizens and organizations could be a strong positive element for society as whole.
- The attempt through the lens of data to better understand the state of wellbeing and needs of specific populations in order to design and produce services that come with genuine positive impact, was commended. The data policy and privacy issues that come with this were seen, however, as a matter to be looked into very closely.

As the program includes all short, medium and long-term objectives, it has been difficult to understand the overall picture. One of the key strategic questions has been whether the AuroraAI program's emphasis is macroeconomic (a better national economy through improved wellbeing and individually empowered citizens) or ultra-individualistic (AI helps you achieve your own life goals, whatever they may be). At the time of writing this article, this was not yet very clear.

### Self-Empowerment Through DigiMe and Data-Managed Life

The DigiMe, or a “holistic 360° profile,” is essentially a digital profile or mirror, composed of certain data, attributes and their values of an individual and their situation in life. The DigiMe centers around a group of eight parameters based on the “The Stiglitz Model” eight-point list of wellbeing factors introduced by Stiglitz et al. (2009). These are: (i) Material living standards (income, consumption, and wealth); (ii) Health; (iii) Education; (iv) Personal activities including work; (v) Political voice and governance; (vi) Social connections and relationships; (vii) Environment (present and future conditions); and (viii) Insecurity, of an economic as well as a physical nature.

Stiglitz et al. (2009, p. 7) argue that these dimensions should be measured to gain insights into the socio-economic realities people live in. They frame this in the context of policies: “In effect, statistical indicators are important for **designing and assessing policies aiming at advancing the progress of society**.”

In the AuroraAI program, however, the eight dimensions are used not only to measure the wellbeing of populations and clusters but also those of individuals. A person's 360° profile in the DigiMe program would then consist of various data sources reflecting the “Stiglitz Model”: health, education, work, income, social connections. It may be argued that this kind of use probably was not the intention of Stiglitz et al. who specifically mentioned these factors as elements to be noted when formulating **policies** that aim to enhance people's quality of life.

**Empowerment** has also been noted in the Board as a slightly problematic concept. In the AuroraAI case the concept seems to refer to a particular chain of events or actions that runs approximately as follows:

*I share my personal attributes with the Aurora Platform, using the DigiMe program that employs the “Stiglitz dimensions” in the background. I receive AI-generated wellbeing estimates and service recommendations in return. I use the recommended services to improve my wellbeing (or to attain a life goal, which may be the same thing). I become better empowered as I learn to control my life through data as I reflect on the contents of the DigiMe service.*

*The AuroraAI catchphrases “Let Your Digital Twin Empower You” and “become the data manager of your life” have been used in the program's rhetoric*.

It should be noted that service recommendations may not be based solely on the user's attributes; they would add the aggregate data of the user's reference cluster(s) the AI has identified to the mix. In this sense, the recommendation logic is quite similar to that of streaming media platforms: it combines your history and features with that of people it assumes are like you in certain critical respects. It remains unclear how the cluster data will be collected, what the data update frequency for that data would be and how a cluster's profile (aggregated attributes) would function for people whose profile places them, data-wise, at the outer edges of the cluster and makes them effectively anomalous in comparison to the people at the cluster's center.

### Effects on Real World Service Provision

One specific point the Board has noted are the “trigger criteria” of recommendations. Young people and their services are a priority target group, especially in the early stages of AuroraAI. In many cases, low threshold mental health and social support services are relevant and valuable for the young. However, these very services in Finland are often quite congested and under-resourced, with sometimes lengthy queues and waiting periods that can stretch to weeks and even months.

If the trigger criteria are set too low, the recommendation engine might recommend these services to a young person going through normal “teenage pains” and who has not considered seeking professional help before. If a state-owned AI directs people like this to these services, an already difficult service situation may become notably worse.

### Use and Sharing of Personal Attributes in the Aurora Platform

The Board has noted that the service recommendation mechanism hinges on people's willingness and ability to either answer questionnaires on their situation, or wellbeing, to describe their situation or personal attributes in natural language, or to share their data from registers (this requires informed consent by the user).

All these actions require different capabilities, ranging from linguistic ability to understanding what the sharing of personal information to a multi-actor network (Aurora Platform) means. With the DigiMe concept, it is somewhat unclear whether it will be used as an application, a user experience (UX) element, or an invisible background element that stores and uses the person's attributes (profile management). Either way, the user should be aware of it and its contents, logic and role in the process which includes personalized wellbeing estimates and service recommendations.

The required ability to understand the overall algorithmic concept and working logic and critically consider the service recommendations may turn this into a service likely to benefit the more capable while leaving the digitally disenfranchised and some minorities by the wayside. Things become more complicated if we assume that the artificial intelligence in AuroraAI is largely another black box whose generated recommendations and estimations, let alone predictions, are not transparent and remain difficult to fully explain.

The questions of **transparency** and **control** also become immediate in the context of the shared personal attributes. What tools will the user have to be able to track the use of their data? Will the users have access to the full list of service providers connected to the Aurora Platform? Can a user block a particular service provider from receiving their data? Can a user see which organizations and companies currently have that user's data on the platform?

In short, the Board is asking how ordinary people can monitor the use of their data in the Aurora Platform and how they can understand why certain services are recommended to them.

### The AuroraAI Account, Profile Management, and Anonymity

In the early AuroraAI scenarios, the users were totally anonymous actors who might only be identified using IP addresses, especially if they returned for service recommendations repeatedly from the same IP. Strong and protected anonymity was taken as a value that creates **trust** and avoids **privacy and security risks**.

Further on, however, the notion of user profiles and profile management came up and, even later, the concept of an AuroraAI account. The Board believes that with these steps, the issues of privacy and anonymity must be brought up and analyzed from scratch as the notion of automatically guaranteed anonymity, if there ever is such a thing, has become critically compromised.

The reasons for setting up profile management and the account arise from the scenario of data-based improvement of wellbeing: if there is no way to follow up users' states of wellbeing after they have received service recommendations based on their attributes given at a certain moment, the AI cannot utilize machine learning to generate better and more accurate recommendations and, eventually, predictions. It needs data points from individuals, albeit anonymous users over time, preferably from a mass of users since data volume is of the essence.

Without this capability, the system would basically be just a smart service search, or assistant, that serves one person at a time in their unique situations and then totally forgets about them. This would give no input for machine learning and AuroraAI would not learn how the recommendations have affected the wellbeing of the user. Ergo, it needs to store user attributes, events and feedback in an account with profile management, which then would most likely be at the heart of the DigiMe solution/service.

**Ensuring the anonymity of users** should be an essential part of the ethical use of AuroraAI. In the AuroraAI program, time periods covering even decades of a person's profile data have been mentioned in the vision of AuroraAI evolving into a personal “life guide” that learns from a person's attributes and events history over time and can thus generate a very accurate and timely guidance, service provision and predictive/proactive recommendations. It is therefore essential that particular attention is paid to this aspect from the anonymity and privacy perspectives.

### Civic Engagement, Inclusion, and Informing the Public

The interests, values, and perspectives of citizens is essential (Levi and Stoker, 2000; Owen et al., 2013) in governmental actions related to AI. Governments should foster and facilitate societal discourse on the **desirability** of AI, and include active **participation** of various stakeholders and citizens. In reciprocal governance, AI experts should take the time to listen to and learn from users, especially their informal and emotional views on how the new service solution differs from existing (non-AI) arrangements, and what is expected of it. User involvement at every stage of the design process is therefore recognized as essential in public sector projects in Finland. However, in AuroraAI, this involvement has not been implemented.

With the exception of NGO representatives on the Ethics Board, there is no information on the abilities different population groups may have in terms of operating within this kind of process. AuroraAI's current focus is on two issues: (1) carrying out the first phase of tech development of the Aurora Platform and the service recommendation engine, (2) developing operative models for leading the human-centric, data-based service production of the future, and restructuring all public sector service production accordingly. The more ambitious technical parts of the program are for the more distant future. This applies especially to the DigiMe concept, and the automatic, personal service recommendations and the human-machine interaction's effects on service production and society in general.

It is argued by the program that, as this long-range vision may easily take a couple of decades to realize, assuming the world does not see a massive paradigm shift in the meanwhile, it makes no sense to bring these high concepts to citizens for discussion and evaluation now. This is a very pragmatic argument and can be defended from that perspective. However, as the current developments, both technical and non-technical, are actually rungs on a ladder that rises toward the eventual vision, it could well be counter-argued that presenting that vision to the public is necessary to justify and measure civic acceptance for the currently ongoing work, as the work is taking place precisely because of the overall vision and not separate from it. As the overall vision is deeply transformative and represents a new socio-technological paradigm, it needs to be accepted as legitimate by civil society before it is widely realized.

## Discussion

The AuroraAI program is striving toward a human-centric, AI-powered society with predictive capabilities and a re-created public sector as the ultimate goal. Thus, the value base of the AuroraAI program is centered on the idea of **a human-centric AI society**: a world in which the public sector and other service providers are aware of the actual real-time needs, challenges and wellbeing of citizens. This society would

- utilize AI and other advanced technologies to empower people individually (via the DigiMe program) to achieve their life goals,
- offer relevant services and entire ecosystems in real time or proactively to both population clusters and individuals to support wellbeing, collective and individual, and
- steer and plan service production, combining all sectors, to respond to people's actual needs, based on all available data on people and population segments.

As such, AuroraAI can be seen as a **socio-technological utopian vision** where artificial intelligence liberates people, enhances wellbeing, boosts the impact of services and drives their cost-effectiveness. This vision, as enticing as it is, comes with a lot of questions and ethical, also legal, puzzles. However, none of this happens suddenly and the current and first baby steps stages of the program may produce lower-level outcomes that may themselves prove useful:

- **A smarter, semantically boosted service search engine**, enriched by a dash of information on the user, can beat Google in search accuracy and practicality. Even if the service recommendation engine is never implemented at full scale, the work, and research put into it will produce progressive results.
- Because people must be able to communicate their information to the Aurora Platform, a UI is needed and chatbots have been picked as the relevant component. The program will produce a generic **AuroraAI chatbot component** that organizations can customize and use in their own services. This is a big plus as it reduces the need for every organization to have their own unique chatbot, and it also assures the bots are ready to communicate with each other.
- The concept of the **life event ecosystem** has been floating around not only in Finland but in a few other countries as well. If the AuroraAI program can come up with a practical solution to technically, legally, and administratively bring together service providers to build largely automatized service networks for life events, it would be a big step forward in making citizens' lives easier.
- The principle of **planning and leading services and ecosystems development and production by human-centric data** is in itself both logical and commendable. If realized in a manner that preserves privacy and individual autonomy and does not discriminate, it could result in a more streamlined and effective service production. This, however, is not a given and there are some serious issues to clarify and ascertain.

These goals would be high enough for any AI project to tackle within one program. However, as AuroraAI moves beyond these challenges and into the realm of individual self-empowerment through data and predictive/proactive personal service recommendations, things get more complicated. Dilemmas arise around not only **profiling, data policies, minorities, and equality** but also **human autonomy, self-rule, the imbalance of power, accountability and transparency, and explicability**. Although various guidelines, codes, or declarations guide the ethical dimension of the implementation and the system to be produced, current AI ethical principles are each an actor's or network's own reaction to this set-up where many unfinished and unresolved issues create opportunities for both completely unintentional negative consequences and deliberate misconduct. **Trust**, **trustworthiness**, and **desirability** in relation to AuroraAI play a significant role here. Of particular interest are also the questions concerning **information management** for one's own wellbeing.

Even if all these aspects are carefully considered, we cannot rely on AI to explain **the dynamics of human life**. Instead, we need to know and understand these issues in order to be able to build the AI system. AI will not free us from understanding the complexities of human life. On the contrary, we should understand them well in order to explain and teach them to the AI machine. This is a tall order given the lack of AI literacy among the public. To move toward this goal, **AI literacy needs to be built among the public, the users of the services, as well as the developers and those who deploy it**. Long and Magerko (2020), define AI literacy as “a set of competencies that enables individuals to critically evaluate AI technologies; communicate and collaborate effectively with AI; and use AI as a tool online, at home, and in the workplace (p. 2)” and outline a range of competencies and design considerations as the basis for building that skillset. Ng et al. (2021) have reviewed the current literature and outlined four aspects of AI literacy: knowing and understanding AI (i.e., know the basic functions of AI and how to use AI applications in everyday life ethically); applying AI (i.e., applying AI knowledge, concepts, and applications in different scenarios); evaluating and creating AI including higher-order thinking skills (e.g., evaluate, appraise, predict, design with AI applications); and AI ethics (i.e., human-centered considerations such as fairness, accountability, transparency, and ethics).

Thus, one can perfectly reasonably argue for a society that has robust, data-based insights into populations, regions, and phenomena and develops certain structures and functions around those insights. However, when the powers that use technologies to gain insights into individuals and their lives for the purpose of understanding or knowing them better, we need to ask: what kind of power do they then wield? This question must be analyzed, discussed and debated, hard, before we can decide if the rewards that may await at the end of the road are indeed worth taking the risk.

Research literature catalogs both positive and negative outcomes in public services from AI use (Eubanks, 2018). Kuziemski and Misuraca (2020) suggest that the first wave of AI innovation will focus on reducing costs (including speeding up and improving digital accuracy). This also seems to have been the ultimate goal of the AuroraAI development. Although there is an emphasis on human-centeredness and with it citizen participation and development in the rhetoric, this aspect has not been addressed much in practice.

One way to involve citizens in the debate on AuroraAI could be to use the so-called “citizen technology.” It could perhaps provide new practical tools for the governance debate on AI, which could help to build common understandings in civil society. Such an information and knowledge infrastructure and “collective-centricity” for the development of networked democracy could accelerate discussions and debates to ensure that they are more meaningful and lead to collective decisions that form the basis for action strategies among multi-level groups of people (Raikov, 2018). There is already evidence that AI-labeled technologies, in combination with other information and communication technologies (ICTs), can promote deliberative and participatory decision-making (Savaget et al., 2019; Arana-Catania et al., 2021). AI tools can potentially improve democratic processes and increase democratic responsiveness and accountability if they are aligned with social and political changes and values that support change (König and Wenzelburger, 2020).

Finally, **timing is critical** in ethics deliberation. The AuroraAI Ethics Board was set up quite late, considering the program's 3 year history at that point. More specifically, as the concept, vision, and planning for technical solutions have been mostly set already, it is questionable whether the Board will be able to have an impact on the program, especially its aims and goals and values, and there are openly expressed suspicions of ethics washing.

All in all, the work of the Ethics Board has proven not only to provide ethical solutions, but has also served as a **learning tool** providing ethical thinking and discussion in a multidisciplinary and multidisciplinary group which has enabled shared learning that would otherwise not be possible.

## Author Contributions

Order of authorship reflects the relative extent of contribution. All authors contributed to manuscript revision, read, and approved the submitted version.

## Funding

JL and NW wish to acknowledge the project Ethical AI for the Governance of the Society (ETAIROS), funded by the Strategic Research Council at the Academy of Finland. AJ's work was supported by a Fulbright-Nokia Distinguished Chair award and a U.S. NSF Awards#1937950, 1939105; USDA/NIFA Award#2021-67021-35329. Any opinions, findings, and conclusions or recommendations expressed in this material are those of the authors and do not necessarily reflect the views of the funding agencies.

## Conflict of Interest

The authors declare that the research was conducted in the absence of any commercial or financial relationships that could be construed as a potential conflict of interest.

## Publisher's Note

All claims expressed in this article are solely those of the authors and do not necessarily represent those of their affiliated organizations, or those of the publisher, the editors and the reviewers. Any product that may be evaluated in this article, or claim that may be made by its manufacturer, is not guaranteed or endorsed by the publisher.

## Footnotes

^1^RSA's Forum for Ethical AI Report, “Artificial Intelligence: Real Public Engagement”: https://www.thersa.org/reports/artificial-intelligence-real-public-engagement.

^2^Involve, “What Does Meaningful Public Engagement Look Like on AI and Ethics?” https://www.involve.org.uk/our-work/our-projects/practice/what-does-meaningful-public-engagement-look-ai-and-ethics.

^3^EGE European Group on Ethics in Science and New Technologies, Statement on Ar-tificial Intelligence, Robotics, and 'Autonomous' Systems. Available onlne at: https://ec.europa.eu/research/ege/pdf/ege_ai_statement_2018.pdf.

^4^The assessment list on trustworthy artificial intelligence. Available online at: https://futurium.ec.europa.eu/en/european-ai-alliance/pages/altai-assessment-list-trustworthy-artificial-intelligence.

^5^https://vm.fi/en/auroraai-en; https://vm.fi/en/national-artificial-intelligence-programme-auroraai
